# Pancreatitis associated with immune checkpoint inhibitors: a pharmacovigilance analysis based on FDA adverse event reporting system (FAERS) database

**DOI:** 10.3389/fphar.2025.1635372

**Published:** 2025-09-22

**Authors:** Lingli Huang, Haitian Wang, Nan Wu

**Affiliations:** ^1^ Department of Pharmacy, Jiangsu Cancer Hospital, Jiangsu Institute of Cancer Research, The Affiliated Cancer Hospital of Nanjing Medical University, Nanjing, Jiangsu, China; ^2^ Department of General Surgery, Jiangsu Cancer Hospital, Jiangsu Institute of Cancer Research, The Affiliated Cancer Hospital of Nanjing Medical University, Nanjing, Jiangsu, China

**Keywords:** immune checkpoint inhibitors, pancreatitis, FAERS database, disproportionality analyses, pharmacovigilance

## Abstract

**Background:**

Immune checkpoint inhibitors (ICIs)-related pancreatitis is a rare but serious immune-related adverse event (AEs). This study aimed to investigate the risk and profile of ICIs-related pancreatitis on a real world setting by analyzing the FDA Adverse Event Reporting System (FAERS) data.

**Methods:**

Data were extracted from the FAERS database from the first quarter of 2011 to the third quarter of 2024. Descriptive analysis was used to represent the clinical features, while reporting odds ratio (ROR), proportional reported ratio (PRR), the Bayesian confidence propagation neural network (BCPNN) and the multiple Gamma Poisson Shrinker (MGPS) were used for disproportionation analysis. The time to onset (TTO) was determined by calculating the interval between pancreatitis AEs and drug initiation time.

**Results:**

A total of 1166 cases with positive signals for ICIs-related pancreatitis were screened, involving atezolizumab, durvalumab, avelumab, tislelizumab, pembrolizumab, nivolumab and ipilimumab. There were significant differences in the distribution of gender, weight, age, reporter, reporting country among all ICIs (*P* < 0.001). As for outcomes, 162 (14.1%) patients died. Avelumab had the highest incidence of death. The results of all four algorithms were consistent, indicating a statistically significant association between overall ICIs and the risk of pancreatitis (ROR 2.44, 95%CI 2.30 - 2.58; PRR 2.43, χ^2^ 979.71; EBGM 2.43, EBGM05 2.29; IC 1.28, IC025 1.22). The ICIs with the highest risk of developing pancreatitis were durvalumab, tislelizumab and avelumab. Avelumab has no significant correlation with pancreatitis in female and patients <65 years old, while other ICIs showed a correlation with pancreatitis, regardless of gender and age. For 491 reports which TTO data were available, the median TTO of ICIs-related pancreatitis was 59.0 days. The TTO of pancreatitis caused by each ICI was statistically significant (*P* = 0.0029). Ipilimumab had the shortest TTO of 37.5 days, while tislelizumab had the longest TTO of 146.5 days. The stratified analysis by gender and age showed that there was no significant difference in TTO.

**Conclusion:**

ICIs may have a significant association with the occurrence of pancreatitis. In clinical applications, it is necessary to closely monitor the indicators related to pancreatitis in patients, such as abdominal pain, nausea, vomiting, elevated serum amylase or lipase, and take timely intervention measures to reduce the risk of complications.

## 1 Introduction

Immune checkpoint inhibitors (ICIs) work by alleviating the inhibited anti-tumor immune response within tumor area and triggering the activation of effector T cells, thereby eliminating the tumor cells. Nevertheless, when the immune response becomes abnormally strengthened, it may also target on normal tissues or cells, thereby precipitating immune-related adverse events (AEs) (Naidoo et al., 2015). Pancreatitis is not common between immunotherapy-related toxicities. Among patients receiving ICIs treatment, the incidences of all-grade pancreatitis and grade ≥ 3 pancreatitis are 0.93% and 0.68% respectively (Zhao et al., 2023). However, in recent years, reports of ICIs-related pancreatitis have gradually increased. The symptoms of ICIs-related pancreatic toxicity can present similar to those of typical acute pancreatitis, including abdominal pain (the most common symptom), back pain, nausea, vomiting, diarrhea, fever, etc. In severe cases, the condition can be life-threatening (Ueno et al., 2021).

As ICIs becoming one of the most cutting-edge treatment in the cancer treating area (Han et al., 2020), the impact of ICIs-related Pancreatitis might further expand. Once ICIs-related pancreatitis happens, according to the Guidelines (Thompson et al., 2024; Haanen et al., 2022), active interventions including hormone treatment and symptomatic treatment in accordance with the diagnostic is recommended. For moderate acute pancreatitis, immunotherapy needs to be suspended, while for patients with severe pancreatitis ICIs need to be permanently discontinued. In these conditions, patients may face not only prolonged inpatient duration, but also impacts on further treatment strategies. Therefore, there exists steroid dependent pancreatitis who encounter with difficulty in tapering glucocorticoids (Kramer et al., 2023). In addition, there remains a probability of recurrence after the cure of pancreatitis, which also poses a challenge to the quality of life of survivors (Thompson et al., 2024). In the context of clinical application, the morbidity of ICIs-related pancreatitis may increase with factors including the combination of ICIs, primary tumor type and a history of previous pancreatitis (George et al., 2019; Zhang et al., 2022), making the condition in real world even more complicated.

The FDA Adverse Event Reporting System (FAERS) database has been standing out as a valuable signal mining tool for it collected a large amount of AE reports from clinical practice, which provide valuable clues for the incidence, risk factors, and clinical characteristics of the AEs. For example, based on FAERS data, it has been found that the application of ICI in solid tumors is associated with increased incidence of all grades of pancreatitis, especially for ICIs combinations (Zhang et al., 2022). Although some studies have preliminarily explored the epidemiology and clinical characteristics of ICIs-related pancreatitis, there is still a lack of large-scale prospective studies focusing on this potential AE. In the present study, we aimed to investigate the risk and feature of ICIs-related pancreatitis on a real world setting by analyzing the FAERS data. The results may enrich the evidence basis for medical staff to early detect the signs for ICIs-related pancreatitis especially in specific populations with high-risk factors.

## 2 Methods

### 2.1 Data sources

We performed a retrospective pharmacovigilance study using data from the FAERS database from the first quarter of 2011 to the third quarter of 2024 (https://fis.fda.gov/extensions/FPD-QDE-FAERS/FPD-QDE-FAERS.html). The FAERS database is a large-scale, comprehensive, and free to the public AE database that collects AE reports from January 2004 to present. This database, which covers AE information for drugs and biologics, as well as medication errors, consists of multiple data tables, mainly including patient demographics and administrative information (DEMO), drug information (DRUG), reaction information (REAC), patient outcomes (OUTC), report sources (RPSR), and drug therapy start and end dates (THER).

### 2.2 Data extraction

In this study, we selected the following ICIs with positive signals for AEs, including programmed death-1 ligand (PD-L1) inhibitors (atezolizumab, durvalumab, avelumab), programmed death-1 (PD-1) inhibitors (tislelizumab, pembrolizumab, nivolumab), and Cytotoxic T - Lymphocyte Antigen 4 (CTLA-4) inhibitor (ipilimumab). These ICIs were classified into primary suspected (PS), secondary suspected (SS), concomitant (C), and interacting (I) according to their role codes in the event. This study focused on ICIs as PS associated with pancreatitis AEs (Meng et al., 2024). Duplicate reports were removed using the PRIMARYID, CASEID, and FDA_DT fields to retain the latest data records. AE reports were coded using preferred terms (PTs) from the Medical Dictionary of Regulatory Activities (MedDRA; version 25.0). This study aimed to identify AEs related to the onset of pancreatitis in patients receiving ICI therapy. PTs were classified using MedDRA’s Systematic Organ Classification (SOC) for more systematic analysis of AEs.

### 2.3 Data analysis

Descriptive analysis was used to represent the clinical features of ICIs-related pancreatitis in the FAERS database. Median (IQR) values were used for continuous variables, and count and percentage values were used for categorical variables. The time to onset (TTO) was determined by calculating the interval between pancreatitis AEs and drug initiation time. Chi-square test was used to analyze the differences in the distribution of gender, weight, age, reporter, reporting country among the various ICIs. A variety of statistical methods including reporting odds ratio (ROR), proportional reported ratio (PRR), the Bayesian confidence propagation neural network (BCPNN) and the multiple Gamma Poisson Shrinker (MGPS), were used for disproportionation analysis (Meng et al., 2024; Zou et al., 2024). The equations and criteria for the four algorithms are shown in Table 1. The signal intensity was calculated by the above method, and the PT with statistical significance was selected. Generally, the higher the algorithm value, the more obvious the signal, indicating the greater degree of association between the target drug and the target adverse reaction. Subgroup analyses were conducted to examine the signal strength and TTO based on gender (male and female subgroup) and age (<65 years and ≥65 years subgroup). The cumulative incidence of no pancreatitis was estimated using the Kaplan-Meier, and the differences among different ICIs were compared through the log-rank test. The ggplot2 R package was used to visualize data in the form of bar charts, forest plots and point range plots. All statistical analyses were performed using R software (version 4.4.1).

**TABLE 1:** Four algorithms used for signal detection

Algorithm	Equation	Criteria
ROR	ROR = (a/b)/(c/d)	95% CI lower >1, a ≥ 3
PRR	PRR = [a/(a + c)]/[b/(b + d)]; χ² = ∑[(O-E)2/E]; (O = a, E = (a + b)(a + c)/(a + b + c + d)	PRR ≥ 2, χ² ≥ 4, a ≥ 3
BCPNN	IC = log2a(a + b + c + d)/[(a + c)(a + b)]	95% CI lower (IC025) > 0
MGPS	EBGM = a(a + b + c + d)/[(a + c)(a + b)]	95% CI (EBGM05) > 2, a > 0

ROR: reporting odds ratio; PRR: proportional reported ratio; BCPNN: Bayesian confidence propagation neural network; MGPS: multiple Gamma Poisson Shrinker; EBGM: empirical Bayesian geometric mean; CI: confidence interval; a: the number of reports containing both target drug and target adverse drug reaction; b: the number of reports containing the target adverse drug reaction with other medications (except the target drug); c: the number of reports containing the target drug with other adverse drug reactions (except the target events); d: the number of all reports; O: observed counts; E: expected counts.

## 3 Results

### 3.1 Descriptive analysis

**FIGURE 1:**
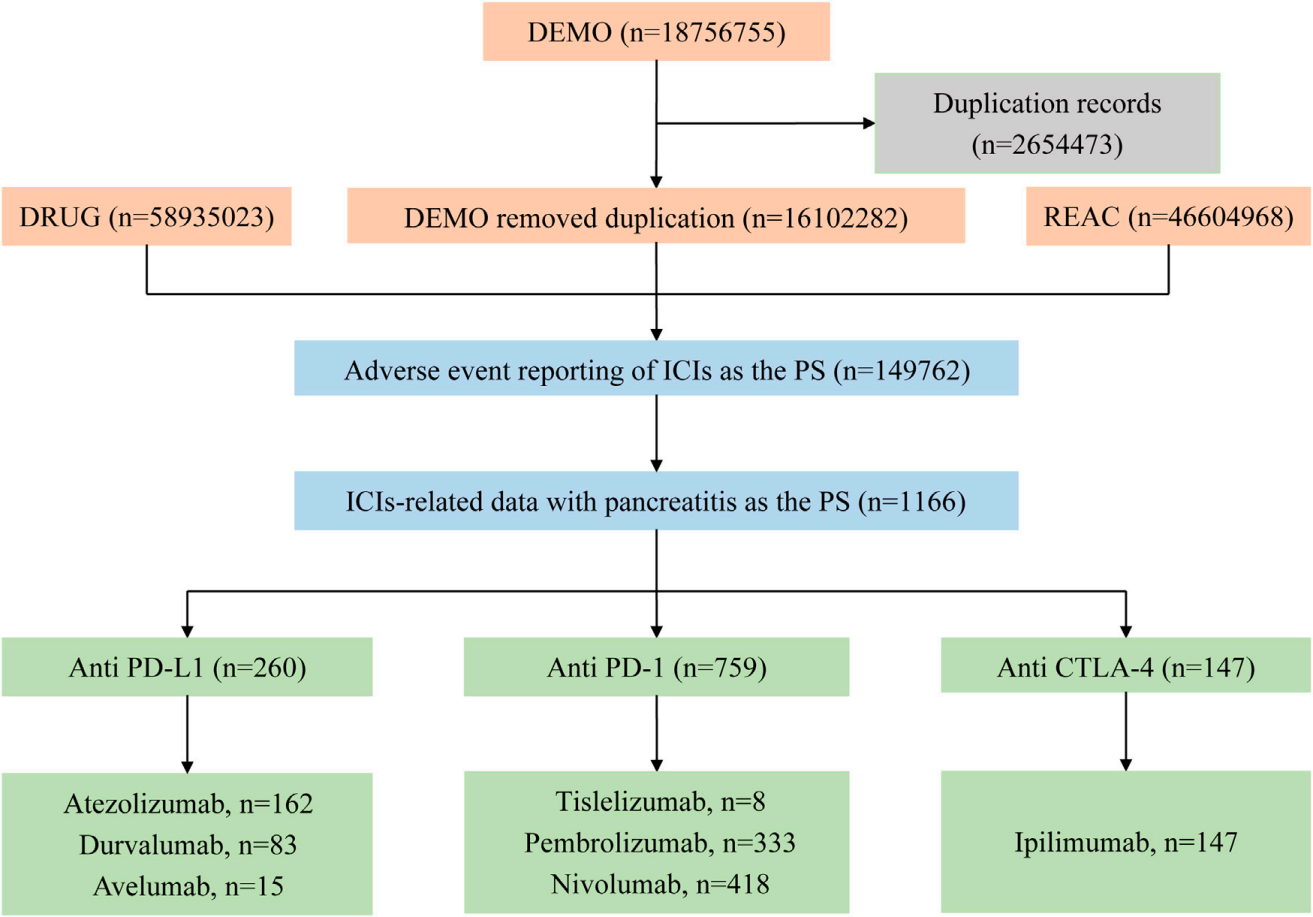
Flow chart of the selection process of pancreatitis caused by immune checkpoint inhibitors in the FAERS database.

Through the search and analysis of the FAERS database, there were a total of 18,756,755 ICIs-related AE reports, of which 149,762 were classified as PS after eliminating 2,654,473 duplicate reports. We ended up screening 1166 reports of ICIs-related pancreatitis, which accounted for only about 0.8% (1166/149762) of ICI AEs. These included 260 cases of PD-L1 inhibitors, 759 cases of PD-1 inhibitors, and 147 cases of CTLA-4 inhibitors. Among them, nivolumab and pembrolizumab had more cases (418, 333), while tislelizumab and avelumab had lower cases (8, 15). The reported cases of atezolizumab, durvalumab and ipilimumab were 162, 83 and 147, respectively. The selection process is shown in Figure 1.

The clinical characteristics of 1151 patients with ICIs-related pancreatitis were shown in Table 2. Among all ICIs, the incidence of ICIs-related pancreatitis was higher in men (621, 54.0%) than in women (416, 36.1%), and males had a higher incidence in each type of ICI. The vast majority of the weight values are missing (733, 63.7%), and most patients weighed 50–100 kg in cases of known weight. Most patients were between 18 and 65 years old. Most pancreatitis AEs were reported by physicians (611, 53.1%), followed by health professional (197, 17.1%), consumer (159, 13.8%), pharmacist (52, 4.5%), and lawyer (1, 0.09%). In terms of reporting countries, most cases were in United States (372, 32.3%), followed by Japan (330, 28.7%), Germany (89, 7.7%), France (86, 7.5%), China (29, 2.5%). It was worth mentioning that all pancreatitis AEs for tislelizumab were reported in China. There were significant differences in the distribution of gender, weight, age, reporter, reporting country among all ICIs (*P* < 0.001).

**TABLE 2:** Clinical characteristics of patients with ICIs-related pancreatitis

Characteristics	Atezolizumab	Durvalumab	Avelumab	Tislelizumab	Pembrolizumab	Nivolumab	Ipilimumab	*P*
Total (n)	162	82	15	8	324	413	147	
Gender (n, %)								<0.001
Female	47 (29.0%)	27 (32.9%)	3 (20.0%)	0 (0%)	147 (45.4%)	146 (35.4%)	46 (31.3%)	
Male	71 (43.8%)	49 (59.8%)	11 (73.3%)	8 (100%)	166 (51.2%)	223 (54.0%)	93 (63.3%)	
Missing	44 (27.2%)	6 (7.3%)	1 (6.7%)	0 (0%)	11 (3.4%)	44 (10.7%)	8 (5.4%)	
Weight (n, %)								<0.001
<50 kg	4 (2.5%)	1 (1.2%)	2 (13.3%)	0 (0%)	15 (4.6%)	13 (3.1%)	6 (4.1%)	
>100 kg	4 (2.5%)	0 (0%)	2 (13.3%)	8 (100%)	7 (2.2%)	11 (2.7%)	4 (2.7%)	
50∼100 kg	54 (33.3%)	27 (32.9%)	4 (26.7%)	0 (0%)	73 (22.5%)	131 (31.7%)	52 (35.4%)	
Missing	100 (61.7%)	54 (65.9%)	7 (46.7%)	0 (0%)	229 (70.7%)	258 (62.5%)	85 (57.8%)	
Age (n, %)								<0.001
<18 years	2 (1.2%)	0 (0%)	0 (0%)	0 (0%)	7 (2.2%)	2 (0.5%)	2 (1.4%)	
>85 years	3 (1.9%)	0 (0%)	1 (6.7%)	0 (0%)	1 (0.3%)	1 (0.2%)	1 (0.7%)	
18∼64.9 years	39 (24.1%)	25 (30.5%)	3 (20.0%)	0 (0%)	131 (40.4%)	179 (43.3%)	72 (49.0%)	
65∼85 years	52 (32.1%)	24 (29.3%)	10 (66.7%)	0 (0%)	118 (36.4%)	137 (33.2%)	50 (34.0%)	
Missing	66 (40.7%)	33 (40.2%)	1 (6.7%)	8 (100%)	67 (20.7%)	94 (22.8%)	22 (15.0%)	
Reporters (n, %)								
Consumer	3 (1.9%)	4 (4.9%)	0 (0%)	0 (0%)	89 (27.5%)	43 (10.4%)	20 (13.6%)	<0.001
Health Professional	34 (21.0%)	3 (3.7%)	1 (6.7%)	6 (75.0%)	44 (13.6%)	97 (23.5%)	12 (8.2%)	
Physician	121 (74.7%)	64 (78.0%)	14 (93.3%)	0 (0%)	164 (50.6%)	176 (42.6%)	72 (49.0%)	
Pharmacist	4 (2.5%)	3 (3.7%)	0 (0%)	2 (25.0%)	17 (5.2%)	24 (5.8%)	2 (1.4%)	
Lawyer	0 (0%)	0 (0%)	0 (0%)	0 (0%)	1 (0.3%)	0 (0%)	0 (0%)	
Others	0 (0%)	0 (0%)	0 (0%)	0 (0%)	9 (2.8%)	73 (17.7%)	40 (27.2%)	
Missing	4 (2.5%)	8 (9.8%)	0 (0%)	0 (0%)	0 (0%)	0 (0%)	1 (0.7%)	
Reporting countries (n, %)								<0.001
United States	41 (25.3%)	17 (20.7%)	3 (20.0%)	0 (0%)	114 (35.2%)	145 (35.1%)	52 (35.4%)	
Japan	57 (35.2%)	18 (22.0%)	3 (20.0%)	0 (0%)	111 (34.3%)	85 (20.6%)	56 (38.1%)	
Germany	5 (3.1%)	2 (2.4%)	1 (6.7%)	0 (0%)	21 (6.5%)	51 (12.3%)	9 (6.1%)	
France	9 (5.6%)	8 (9.8%)	1 (6.7%)	0 (0%)	22 (6.8%)	40 (9.7%)	6 (4.1%)	
China	5 (3.1%)	9 (11.0%)	0 (0%)	8 (100%)	3 (0.9%)	4 (1.0%)	0 (0%)	
Other Contries	45 (27.8)	28 (34.1%)	7 (46.7%)	0 (0%)	53 (16.4%)	88 (21.3%)	24 (16.3%)	

**FIGURE 2:**
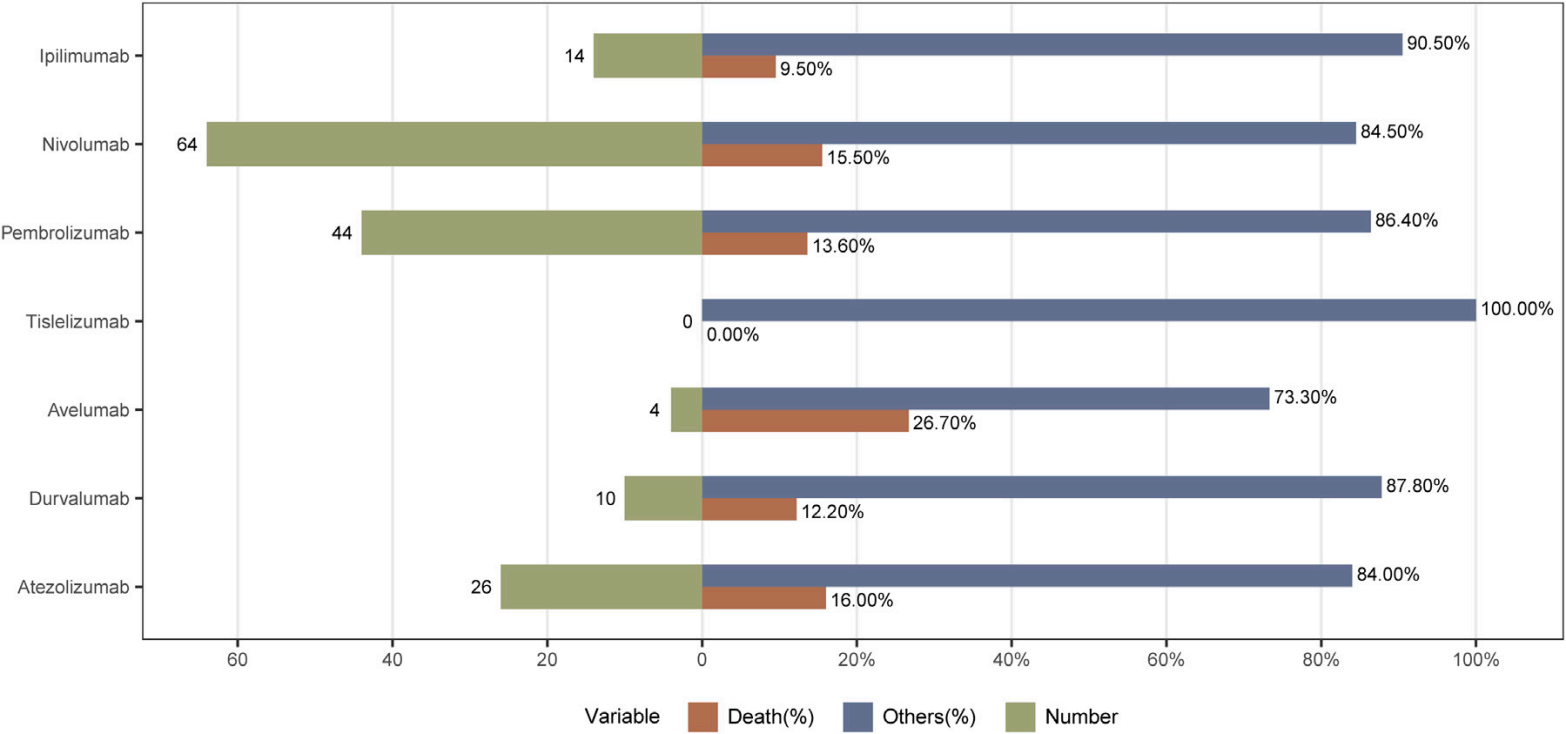
Death cases and their proportion in ICIs concomitantly with pancreatitis.

As shown in Table 3, all ICIs-related AEs basically showed an increasing trend year by year. From 2011 to 2014, the incidence of ICIs-related pancreatitis was relatively low. The pancreatitis AEs increased year by year from 2015 to 2020. Compared with 2020, the reported cases from 2021 to 2023 were decreased. The incidence of pancreatitis caused by ICIs was highest in 2024. As for outcomes, 162 (14.1%) patients died. Avelumab had the highest incidence of death and no deaths associated with tislelizumab was reported. The incidences of death in patients with atezolizumab, nivolumab, pembrolizumab, durvalumab, and ipilimumab were 16.0%, 15.5%, 13.6%, 12.2%, and 9.5%, respectively. The specific outcome is shown in Figure 2.

**TABLE 3:** The counts of patients with ICIs-related pancreatitis yearly from 2011Q2 to 2024Q3

Years	NO_target_PT	target_PT	Overall
2011	281 (0.17%)	2 (0.17%)	283 (0.17%)
2012	1050 (0.64%)	4 (0.35%)	1054 (0.64%)
2013	975 (0.6%)	1 (0.09%)	976 (0.6%)
2014	1769 (1.09%)	7 (0.61%)	1776 (1.08%)
2015	4500 (2.76%)	33 (2.87%)	4533 (2.76%)
2016	9331 (5.73%)	40 (3.48%)	9371 (5.71%)
2017	13222 (8.12%)	76 (6.6%)	13298 (8.11%)
2018	15781 (9.69%)	118 (10.25%)	15899 (9.69%)
2019	18461 (11.33%)	134 (11.64%)	18595 (11.34%)
2020	17579 (10.79%)	161 (13.99%)	17740 (10.82%)
2021	18706 (11.49%)	132 (11.47%)	18838 (11.49%)
2022	21264 (13.06%)	142 (12.34%)	21406 (13.05%)
2023	20882 (12.82%)	126 (10.95%)	21008 (12.81%)
2024	19070 (11.71%)	175 (15.2%)	19245 (11.73%)
Total	162871 (100%)	1151 (100%)	164022 (100%)

### 3.2 Disproportionality analysis

To assess the relationship between ICIs and pancreatitis AEs, ICIs was disambiguated from all other drugs in the FAERS database, and different ICIs had different profiles of pancreatitis (Table 4). The results of all four analyses were consistent, indicating a statistically significant association between overall ICIs use and the onset of pancreatitis (ROR 2.44, 95%CI 2.30 - 2.58; PRR 2.43, χ^2^ 979.71; EBGM 2.43, EBGM05 2.29; IC 1.28, IC025 1.22). Comparing the various types of ICIs to all the other drugs in the analysis, durvalumab had the strongest signal (ROR 3.37, 95%CI 2.71 - 4.18; 3.36, χ^2^ 137.17; EBGM 3.35, EBGM05 2.8; IC 1.74, IC025 1.43), followed by tislelizumab (ROR 3.28 95%CI 1.64 - 6.57; PRR 3.27, χ^2^ 12.64; EBGM 3.27, EBGM05 1.83; IC 1.71, IC025 0.75), avelumab (ROR 3.23, 95%CI 1.95 - 5.37; PRR 3.23, χ^2^ 23.06; EBGM 3.23, EBGM05 2.11, IC 1.65, IC025 0.97), and atezolizumab (ROR 2.84, 95%CI 2.43 - 3.31; PRR 2.83, χ^2^ 191.28; EBGM 2.82, EBGM05 2.48, IC 1.50, IC025 1.27). All algorithms, ROR, PRR, MGPS and BCPNN showed that atezolizumab, durvalumab, avelumab, pembrolizumab, nivolumab, ipilimumab were significantly associated with pancreatitis AEs, but MGPS algorithm found that the association was not strong for tislelizumab (EBGM05 1.83).

**TABLE 4:** Adverse events of different ICIs

Drug	N (a)	ROR(95%Cl)	PRR(χ²)	EBGM(EBGM05)	IC(IC025)
Total ICIs	1166	2.44 (2.30 - 2.58)	2.43 (979.71)	2.43 (2.29)	1.28 (1.22)
PD-L1 inhibitor					
Atezolizumab	162	2.84 (2.43-3.31)	2.83 (191.28)	2.82 (2.48)	1.50 (1.27)
Durvalumab	83	3.37 (2.71-4.18)	3.36 (137.17)	3.35 (2.8)	1.74 (1.43)
Avelumab	15	3.23 (1.95-5.37)	3.23 (23.06)	3.23 (2.11)	1.69 (0.97)
PD-1inhibitor					
Tislelizumab	8	3.28 (1.64-6.57)	3.27 (12.64)	3.27 (1.83)	1.71 (0.75)
Pembrolizumab	333	2.2 (1.97-2.45)	2.19 (214.61)	2.18 (2)	1.13 (0.97)
Nivolumab	418	2.21 (2-2.43)	2.2 (272.67)	2.19 (2.02)	1.13 (0.99)
CTLA-4 inhibitor					
Ipilimumab	147	2.79 (2.37-3.28)	2.78 (167.69)	2.78 (2.43)	1.47 (1.24)

**FIGURE 3:**
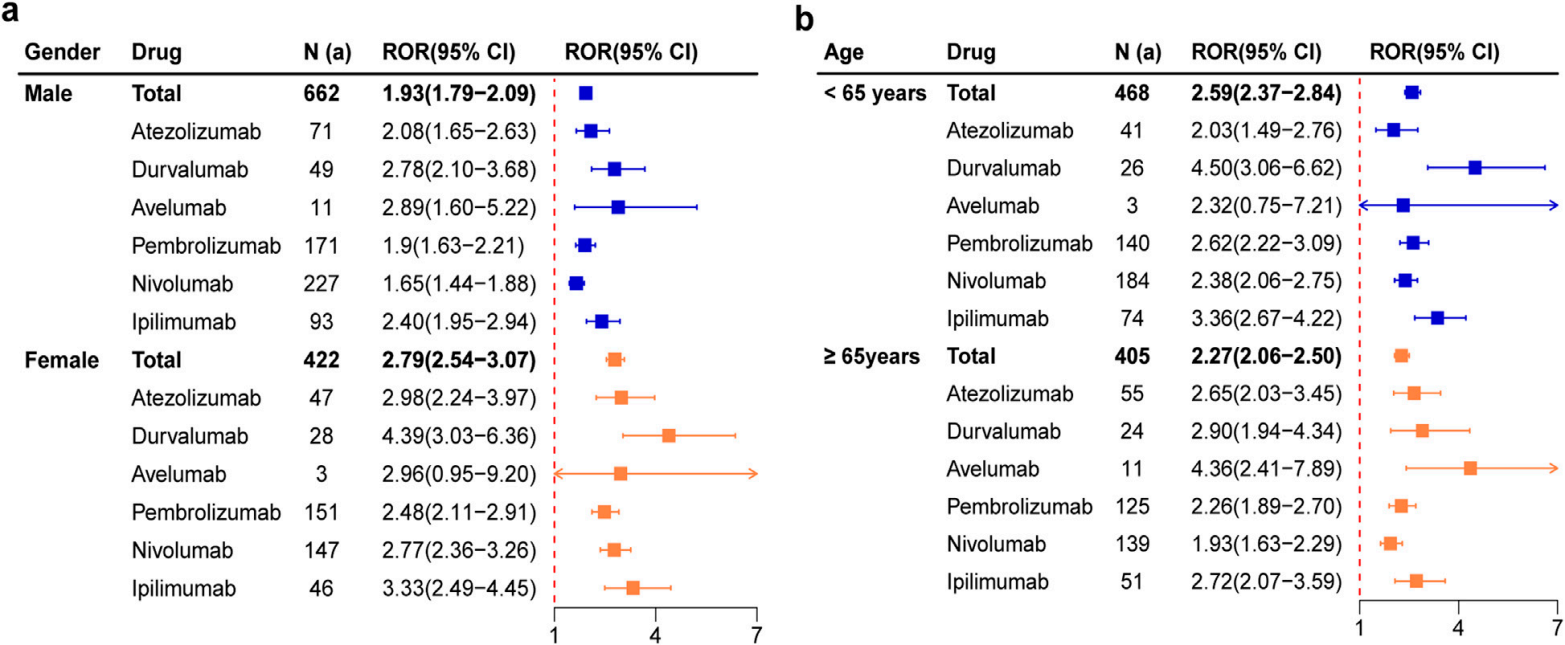
Forest plots of pancreatitis AEs for each ICIs based on subgroup analysis by gender **(a)** and age **(b)**.

Due to the insufficient sample size, tislelizumab was not included in the subgroup analysis. The ROR of six ICIs for male and female were 1.93 (1.79-2.09) and 2.79 (2.54-3.07) respectively, and the signal trend was consistent (Figure 3a). The signals of atezolizumab, durvalumab, pembrolizumab, nivolumab, ipilimumab were more pronounced in female than in male. It was worth noting that Avelumab showed a positive signal for pancreatitis only in males. The ROR of six ICIs for patients < 65 years and ≥ 65 years were 2.59 (2.37-2.84) and 2.27 (2.06-2.50) respectively, and the signal trend was also consistent (Figure 3b). The signals of durvalumab, pembrolizumab, nivolumab and ipilimumab were stronger in patients <65 years, while atezolizumab was more relevant in patients ≥65 years. In particular, avelumab had no significant correlation with pancreatitis in patients <65 years old.

**FIGURE 4:**
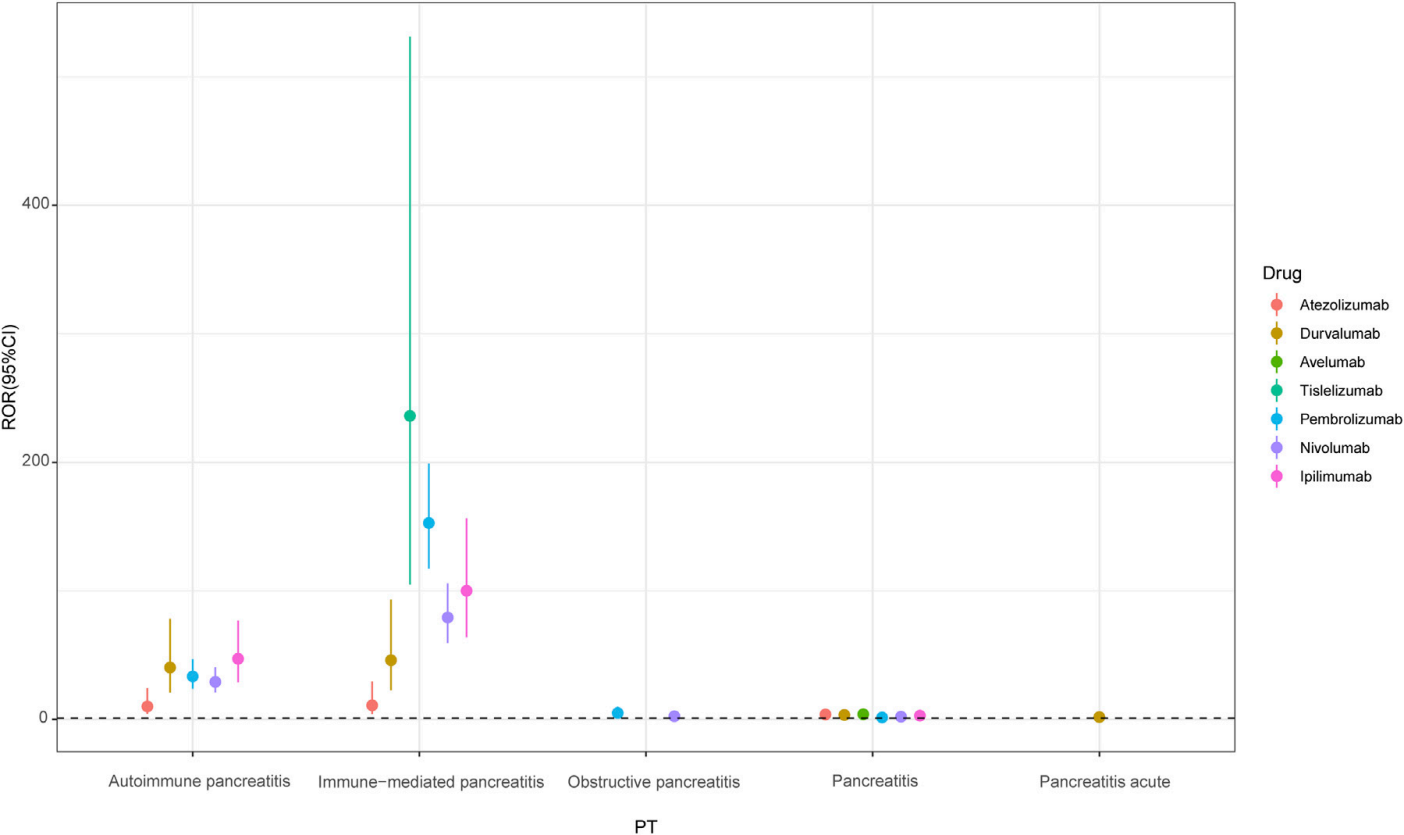
Point range plots of pancreatitis under PT classification of different immune checkpoint inhibitors. PT, preferred term; ROR, reporting odds ratio.

Among the 40 signals unearthed at the PT level, 20 of them were positive signals, as detailed in Supplementary Table S1. The PT signals of durvalumab, pembrolizumab, nivolumab, ipilimumab, atezolizumab, avelumab and tislelizumab involved four, four, four, three, three, one and one positive signal, respectively (Figure 4). Tislelizumab had a signal (ROR 236.12, 95%CI 104.92 - 531.4) of at the PT “immune-mediated pancreatitis” level, the strongest of all the PT’s. Pembrolizumab, ipilimumab, nivolumab, durvalumab and atezolizumab also showed very significant signals at PT “immune-mediated pancreatitis”, with ROR (95%CI) of 152.86(117.38-199.07), 100.1 (63.98-156.59), 79.26 (59.41-105.74), 46.05 (22.76-93.17) and 10.92 (4.06-22.97). At the PT “autoimmune pancreatitis” level, the signal strength of ipilimumab, durvalumab, pembrolizumab, nivolumab, and atezolizumab was also stronger, and the ROR (95%CI) were 47.18 (29.02-76.69), 40.35 (20.79-78.31), 33.44 (23.8-47.0), 29.17 (21-40.53) and 10.05 (4.15-24.3), respectively. Pembrolizumab and nivolumab were found to be associated with obstructive pancreatitis. Apart from tislelizumab, all other ICIs showed positive signals at PT “pancreatitis”. Only durvalumab showed the signal strength at PT “pancreatitis acute”.

### 3.3 TTO analysis

**FIGURE 5:**
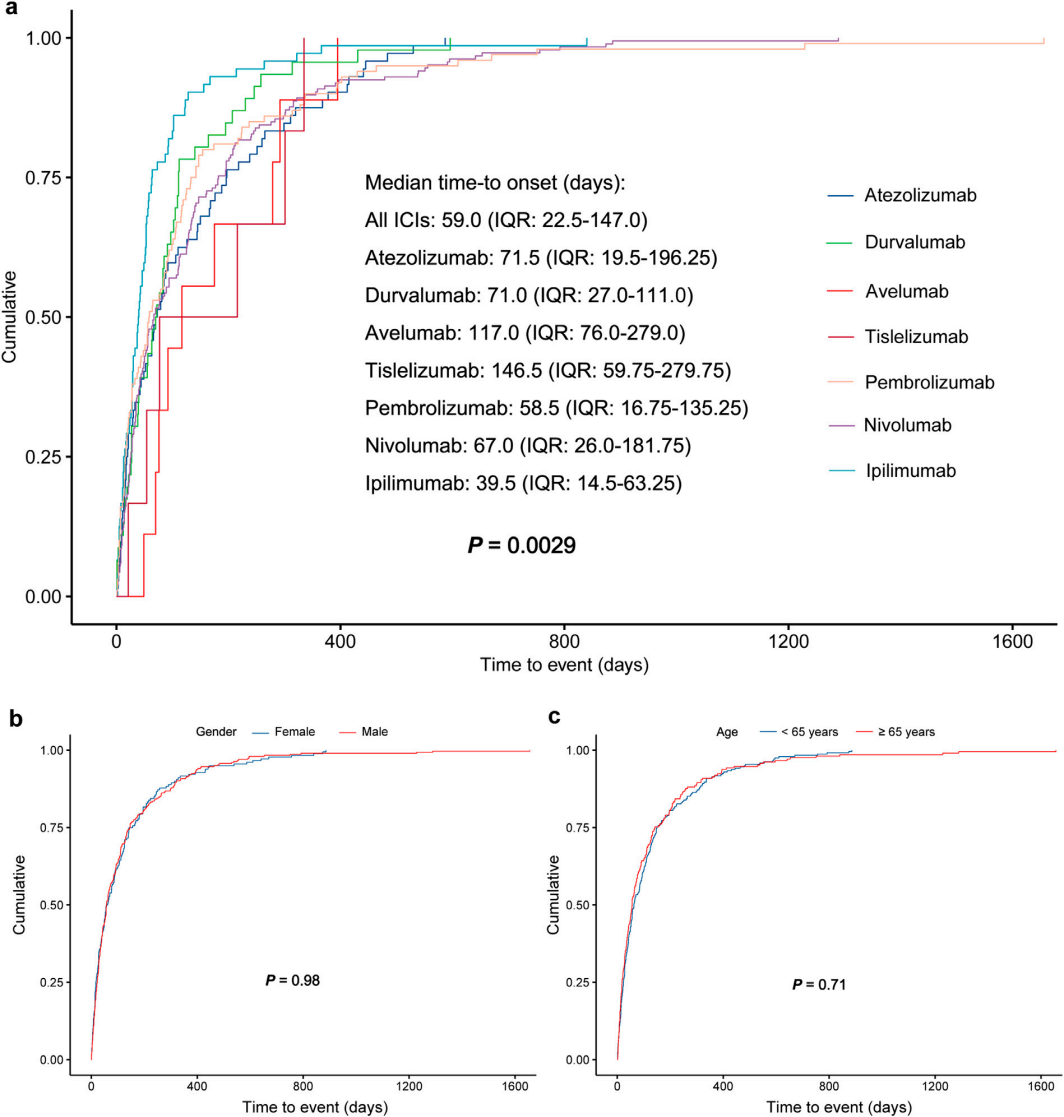
The TTO of pancreatitis AEs for each ICI **(a)**, and the TTO of pancreatitis AEs for total ICIs based on subgroup analysis by gender **(b)** and age **(c)**. TTO, time to onset.

Figure 5a shows the TTO of pancreatitis AEs for various ICIs. For 491 reports which TTO data were available, the median TTO of ICIs-related pancreatitis was 59.0 days (IQR: 22.5–147.0 days). CLTA-4 inhibitor ipilimumab had the shortest TTO of 39.5 days (IQR:14.5–63.25 days), while PD-L1 inhibitor tislelizumab had the longest TTO of 146.5 days (IQR:59.75–279.75 days). The TTO of avelumab, atezolizumab, durvalumab, nivolumab and pembrolizumab were 117.0 days (IQR: 76.0–279.0 days), 71.5 days (IQR: 19.5–196.25 days), 71.0 days (IQR: 27.0–111.0days), 67.0 days (IQR: 26.0–181.75 days), and 58.5 days (IQR: 16.75–135.25 days), respectively. The TTO of pancreatitis AEs caused by each ICI was compared, and the difference was statistically significant (*P* = 0.0029). The stratified analysis of total ICIs by gender and age showed that there was no significant difference in TTO between men and women, and no statistical difference was found in TTO between those >65 years and ≤65 years (*P* > 0.05) (Figures 5b,c). The subgroup analysis results by gender and age for each ICI are shown in Supplementary Figures S1, S2, and no statistically significant findings were observed (*P* > 0.05).

## 4 Discussion

The advent of ICIs has significantly altered the landscape of cancer treatment. At present, no fewer than 16 ICIs are available on the global market. However, the underlying pancreatic toxicity of these compounds remains to be frequently underappreciated. One strategy for enhancing drug safety involves systematic assessment of the potential pancreatic toxicity linked to specific ICIs. Our study provides a comprehensive understanding through a retrospective pharmacovigilance analysis of data from the FAERS database over the past 14 years.

Between 2011 and 2024, along with the expansion of ICIs in various cancer therapies, the number of ICIs-related pancreatitis cases increased from 2 to 175, an increase of over 80 - fold. Those cases provide a basis for in-depth research on the AEs of such drugs in real - world clinical setting. Over 70% of the reports in this study were submitted by healthcare professionals, indicating the high reliability of our findings since immune-related pancreatitis demands professional medical diagnosis and differential. Some cases suffered from severe consequences, which highlights the importance of the management of ICIs-related pancreatitis. Descriptive analysis showed that ICIs-related pancreatitis events were more common in male patients, suggesting the presence of certain potential risk factors worth further exploration. There is a significant imbalance in the geographical sources of ICIs-related pancreatitis reports, with the majority of cases coming from the United States (32.2%), followed by Japan (28.7%), Germany (7.7%), France (7.5%) and China (29, 2.5%). This difference may be related to the accessibility of ICIs and pharmacovigilance practices in different regions. In addition, genetic and environmental factors specific to certain populations may affect the incidence rate of pancreatitis. Meanwhile, such geographical disparities highlight the necessity for globally unified AEs reporting in the future. Four disproportionality-based analysis methods were employed in our study, including ROR, PRR, MGPS, and BCPNN. Among the seven ICIs investigated in this study, the signals of pancreatitis were equally detected by the four analysis methods. The results are congruent with those reported in several extant studies (George et al., 2019; Jia et al., 2020; Rogers et al., 2020; Hamzah et al., 2019) and also closely parallel the information in the drug product labels. This concordance highly validates the reliability of the present study. Significantly, it further elucidates that the induction of pancreatitis is a prevalent phenomenon across diverse ICIs targeting PD-1, PDL-1, and CTLA-4.

To date, there is no clear evidence to predict patients who is predisposed to ICIs-related AEs (Brazel et al., 2025). Based on the existing research data, the risk factors for ICIs-related pancreatitis may include concomitant medication, primary neoplasm, and prior history of pancreatitis (Zhao et al., 2023; Zhang et al., 2022). Multiple studies have reported that the concomitant use of ICIs significantly elevates the risk of pancreatitis (George et al., 2019; Zhang et al., 2022; Lee et al., 2025). A retrospective investigation demonstrated that ICIs-related pancreatitis appeared in 5% cases undergoing combined ICIs therapy, whereas the incidences in patients receiving monotherapy with ipilimumab, nivolumab, and pembrolizumab are 1.1%, 1.9%, and 0.9%, respectively (El Majzoub et al., 2019). A meta-analysis showed in comparison to patients with non-melanoma malignancies, melanoma patients exhibit 50% higher incidence of lipase elevation and grade 2 pancreatitis (George et al., 2019). This meta-analysis also illustrated that the risk of ICIs-related pancreatic toxicity was increased among patients with a prior history of pancreatitis (*P* = 0.013) (George et al., 2019).

In our study, male constituted a higher proportion of those with ICIs-related pancreatitis. The results of the subgroup analysis showed that total ICIs were strongly associated with pancreatitis in both male and female, with a stronger signal observed in female. It should be noted that the use of avelumab in the female population seemed to have no relation to pancreatitis. To date, the impact of gender on ICIs-related pancreatitis remains elusive. Zhang, Y. et al. Observed in their research that male patients accounted for a larger proportion of cases with ICIs-related pancreatic injury, with no significant difference in reporting frequency between males and females (0.30% vs. 0.33%, χ^2^ = 0.690, *p* = 0.406) (Zhang et al., 2022).

Total ICIs showed a stronger signal of ICIs-related pancreatitis in patients <65 years old compared to those ≥65 years old. The signal of durvalumab, pembrolizumab, nivolumab and ipilimumab were stronger in patients <65 years old, while atezolizumab showed a greater association with pancreatitis in patients ≥65 years old. It is worth noting that avelumab did not show a significant association with pancreatitis in patients <65 years old. In a retro-prospective analysis, younger patients are more subject to experience endocrine toxicities (Paderi et al., 2021). Zhang, Y. et al. also reported patients under 65 years of age had a higher rate of pancreatitis than the older group (42.97% vs 35.39%), and a significant difference was noted in the reporting frequency (0.38% vs 0.27%, *P* < 0.001) (Zhang et al., 2022). In another newly published real-world pharmacovigilance study, the median age at ICIs-related pancreatitis diagnosis was 58 years (Fang et al., 2025). However, the association between age and ICI-related AEs may vary depending on the AEs type. In the ELDERS study (Gomes et al., 2021), no significant difference was found in the incidence of AEs grade 3–5 between older and younger cohorts (18.6% versus 12.9%, *P* = 0.353).

As for weight, the majority of patients had a weight ranging from 50 to 100 kg. Interestingly, for the key roles of the immune system and energy metabolism are taking in tumorigenesis, it is emphasized to evaluating body weight prior to the initiation of ICI therapy in cancer patients (Rassy et al., 2018). A meta-analysis has shown that elevated body mass index (BMI) was significantly associated with AEs in patients on ICI therapy, with stable associations across subgroups (Guzman-Prado et al., 2021). However, another retrospective analysis showed no significant difference beweent BMI and ICI-related AEs (Gómez-Banoy et al., 2024). The relationship between body weight and the onset of ICIs-related pancreatitis worth further investigation.

While evidence for predicting factors of ICIs-related pancreatitis remains insufficient, early detecting of pancreatitis presents a challenge. One of the reasons is that, monitoring of pancreatic function has not been recommended before and during the ICIs treatment (Thompson et al., 2024; Haanen et al., 2022). Thus, to ensure timely recognition of AEs, it is imperative that clinicians consistently consider ICIs-related adverse events as a potential etiology for any complication presenting in patients receiving ICIs therapy (Brazel et al., 2025). Within the routine monitoring items recommended by the guidelines, blood glucose may provide indicative value, for it can sometimes present as the first symptom in autoimmune pancreatitis (Jia et al., 2020; Rogers et al., 2020). Otherwise, physical symptoms indicative of pancreatitis encompass epigastric pain, nausea and vomiting, fever, and diarrhea (Hamzah et al., 2019). For obstructive pancreatitis, it may present with jaundice (Ashraf et al., 2021). When the pancreas is visible in patients’ regular CT scans, radiological changes may have value in the detection of ICIs-related pancreatitis (Nakano et al., 2022).

The strongest PT signal in our study appears in immune-mediated pancreatitis, followed by autoimmune pancreatitis. In fact, autoimmune pancreatitis is a special type of immune-mediated pancreatitis. Moreover, ICIs-associated pancreatic injury is now considered to be a form of autoimmune pancreatitis, which is named as type 3 autoimmune pancreatitis (Sayed Ahmed et al., 2022). These high signals suggest a close relationship between ICIs-related pancreatitis and immune imbalance. This aligns with the findings in earlier published case series, wherein ICIs-related pancreatitis exhibited clinical and radiological features closely resembling those of autoimmune pancreatitis (Kramer et al., 2023; Tanabe et al., 2024). The labels of these ICIs only mentioned pancreatitis, and rarely specify the specific type. Remarkably, this study detected some signals that were not explicitly mentioned in the drug labels. There were positive signals for acute pancreatitis in durvalumab, while obstructive pancreatitis was present in pembrolizumab and nivolumab. These PTs, which are listed in parallel with ‘pancreatitis’ in the MedDRA, containing the mechanism or manifestation information, are distinguished from the commonly descried “pancreatitis” within the labels for ICIs. The identification of these signals, on one hand, may be associated with the description of AEs in drug clinical trials. On the other hand, it reveals the diversity of pancreatitis symptoms induced by ICIs in real-world setting, as well as the potential correlations between these symptoms and personal underlying conditions. Therefore, in clinical practice, the broader symptoms of pancreatitis should be closely monitored, and these signals should not be ignored simply because the number of related reports is small.

Analysis on TTO unveils the dynamics of pancreatitis subsequent to the initiation of ICIs therapy. Since ICIs-related pancreatitis is not common, early detection, differential diagnosis and etiological treatment are of paramount significance. Our real-world pharmacovigilance study determined that the median TTOs of different ICIs were within several months after drug administration which is consistent with the reports in the existing literature (Lee et al., 2025; Tang et al., 2021). This temporal information is absent from the drug labels. Nevertheless, it offers clinicians a general time framework, facilitating the development of tailored monitoring regimens for diverse medications. Meanwhile, it should be noticed that there was early-onset toxicity occurring as early as the first day of ICI administration (Jiang et al., 2018). Furthermore, we also observed that in some cases, pancreatitis emerged even 1 year or several years after the initiation of ICIs treatment. Similar late toxicity occurring after 1 year of therapy with ICIs have been reported earlier (Domínguez Bachiller, 2020; Yilmaz and Baran, 2022). Yong et al. reported pancreatitis onset even over 5 years after the initial administration of nivolumab (Pan et al., 2025). This suggests that even several years after the use of ICIs, once symptoms of pancreatitis occur, medical practitioners should be vigilant about the possibility of ICIs-related pancreatitis. Differential diagnosis may be necessary when appropriate. Notably, some late-onset adverse reactions can still be severe. In a pooled study involving 8,436 cases, it was found that for PD-1, PDL-1, and CTLA-4, the onset time of grade 3 or higher AEs was significantly later than that of all AEs (Tang et al., 2021).

Among the seven ICIs, the CTLA-4 inhibitor ipilimumab showed the shortest median TTO. As indicated in the prior research, ipilimumab ranked the worst among all evaluated immune checkpoint inhibitors with the incidence of AEs falling between common and very common (Oliveira et al., 2024). In contrast, other ICIs such as nivolumab and pembrolizumab rank lower, with the incidence of AEs being common (between 1/100 and 1/10) (Oliveira et al., 2024). Moreover, ipilimumab is often used in combination with other ICIs in clinical practice. Currently, ipilimumab has been approved for melanoma, renal cell carcinoma, colorectal cancer, hepatocellular carcinoma, and non-small cell lung cancer. Except for the adjuvant treatment of adult patients with cutaneous melanoma, ipilimumab is recommended to be used in combination with another ICI for the remaining indications. However, multiple studies have already suggested that the combined use of ICIs increases the risk of immune-related adverse reactions (George et al., 2019; Zhang et al., 2022; Oliveira et al., 2024; Hori et al., 2024).

Among all cases with recorded outcomes, the proportion of fatal cases exceeded 10% of the total, Specifically, the mortality rate of patients treated with avelumab was the highest, reaching 27.3% (3/11). In another research involving 61 patients with ICIs-related pancreatitis from 58 studies, most patients received anti-PD-1/PD-L1 monotherapy (78.7%) or combination therapy with CTLA-4 inhibitors (19.7%), with 5 cases of death (accounting for 8.2%) (Fang et al., 2025). The relatively high proportion of fatal appears noteworthy as compared with approximately 0.35% incidence of fatal cases due to overall immune-related adverse reactions among patients receiving ICIs treatment (Wang et al., 2018). The exact mortality rate of patients with pancreatitis induced by ICIs remains unascertained. In a retrospective study (n = 843) conducted in 2024, no deaths related to ICIs-related pancreatitis were reported (Hori et al., 2024). Masayuki et al. reported a case of 62-year-old male patient with unknown primary tumor lesion, who successively experienced immune-related pneumonia and immune-related pancreatitis, after receiving pembrolizumab treatment. Even being successfully treated, another episode of pancreatitis recurred did not show any response to treatments, including high-dose corticosteroids, and the patient ultimately passed away (Ueno et al., 2021). It was also reported that a patient with a history of nivolumab use and prior liver injury eventually died of severe pancreatitis that unresponsive to steroid therapy, although he only had asymptomatic elevated pancreatic enzymes 2 days earlier (Nagao et al., 2024). Although the current guidelines recommend that patients without severe pancreatitis should consider using ICI after improvement of pancreatic injury, it seems appropriate to conduct a careful assessment before challenge again, as further severe pancreatitis can be fatal.

At present, the exact pathogenesis of ICIs-related pancreatitis has not been well elucidated. Pathological changes found in prior studies (Yoneda et al., 2019; Di Giacomo et al., 2009; Liu et al., 2019) provide evidence for the induction of functional T-cell activation in ICIs-related pancreatitis. Furthermore, the tumor microenvironment, immune infiltrate, and gut microbiota may also be potential causes of AEs related to ICIs (Ramos-Casals et al., 2020; Postow et al., 2018).

Although our study highlights the association between ICIs and pancreatitis, there are still several limitations to be considered. Firstly, as a spontaneous reporting system, the FAERS database has multiple inherent issues such as data missing, selection bias, and information sources, so it is unable to comprehensively adjust for confounding factors such as comorbidities and concurrent medications. Further verification is needed through electronic health records or prospective studies in the future. Secondly, the duration for which different drugs have been available on the market and their accessibility across diverse regions also influence the number of adverse reaction cases. Nevertheless, our study has conducted a cautious and reasonable assessment of the risk of pancreatitis associated with several ICIs. The results of our research may greatly enhance the safety and effectiveness of ICIs-based therapies, enabling early detection and management of potential pancreatitis-related complications. It is necessary to enhance the awareness of pancreatitis and conduct close monitoring. Proactive consideration should be given to measures such as intensive monitoring, screening for risk factors, and patient education on medication, especially within the first few months after starting ICI treatment.

## 5 Conclusion

This study confirms the association between ICIs and pancreatitis based on large-scale real-world data, providing crucial drug safety evidence for clinical practice. Female and non-elderly patients (<65 years old) may have a higher risk of developing pancreatitis, but avelumab is only associated with pancreatitis in male and elderly patients (≥65 years old). The TTO of pancreatitis varies significantly among different ICIs (median time 39.5–146.5 days), and is not related to gender and age. It should be noted that ICIs-related pancreatitis induced by ipilimumab mostly occurs in the early stage after medication. During ICI treatment period, abdominal pain, nausea, vomiting, fever, and diarrhea, serum lipase or aminase, should be regularly monitored, and imaging (CT/MRI) should be used for diagnosis. In the future, it is necessary to explore biomarkers (such as autoantibodies) to predict high-risk populations and optimize immunotherapy strategies.

## Data Availability

The original contributions presented in the study are included in the article/Supplementary Material, further inquiries can be directed to the corresponding author.
