# Supplementary material for: Pancreatitis associated with immune checkpoint inhibitors: a pharmacovigilance analysis based on FDA adverse event reporting system (FAERS) database
Source: Front Pharmacol. 2025 Sep 22;16:1635372. doi: 10.3389/fphar.2025.1635372 (PMC12497980; doi:10.3389/fphar.2025.1635372)
Supplement: Supplementary file 1 [file Table1.docx]

Table S1 ICIs-related pancreatitis at the PT level in the FAERS database

| Drugs | Preferred term (PT) | Report number | ROR(95%Cl) | PRR(X2) | EBGM(EBGM05) | IC(IC025) |
| --- | --- | --- | --- | --- | --- | --- |
| Atezolizumab | Pancreatitis | 131 | 3.81 (3.21-4.52) | 3.8 (269.23) | 3.79 (3.28) | 1.92 (1.67) |
|  | Pancreatitis acute | 18 | 1.08 (0.68-1.71) | 1.08 (0.1) | 1.08 (0.73) | 0.11 (-0.56) |
|  | Autoimmune pancreatitis | 5 | 10.05 (4.15-24.3) | 10.05 (40.11) | 9.91 (4.73) | 3.31 (2.12) |
|  | Immune-mediated pancreatitis | 4 | 10.92 (4.06-29.33) | 10.92 (35.44) | 10.75 (4.7) | 3.43 (2.12) |
|  | Pancreatitis chronic | 2 | 1.27 (0.32-5.09) | 1.27 (0.12) | 1.27 (0.4) | 0.35 (-1.32) |
|  | Obstructive pancreatitis | 1 | 1.36 (0.19-9.71) | 1.36 (0.1) | 1.36 (0.26) | 0.45 (-1.6) |
|  | Oedematous pancreatitis | 1 | 2.27 (0.32-16.15) | 2.27 (0.71) | 2.26 (0.44) | 1.18 (-0.87) |
| Durvalumab | Pancreatitis | 51 | 3.46 (2.63-4.56) | 3.46 (88.99) | 3.45 (2.74) | 1.79 (1.39) |
|  | Pancreatitis acute | 13 | 1.79 (1.04-3.09) | 1.79 (4.54) | 1.79 (1.14) | 0.84 (0.07) |
|  | Autoimmune pancreatitis | 9 | 40.35 (20.79-78.31) | 40.33 (335.27) | 39.2 (22.51) | 5.29 (4.37) |
|  | Immune-mediated pancreatitis | 8 | 46.05 (22.76-93.17) | 46.03 (340.87) | 44.55 (24.71) | 5.48 (4.5) |
|  | Pancreatitis chronic | 2 | 2.87 (0.72-11.51) | 2.87 (2.44) | 2.87 (0.9) | 1.52 (-0.15) |
| Avelumab | Pancreatitis | 11 | 3.96 (2.19-7.16) | 3.95 (24.28) | 3.95 (2.41) | 1.98 (1.15) |
|  | Autoimmune pancreatitis | 2 | 47.25 (11.76-189.81) | 47.23 (89.93) | 46.94 (14.66) | 5.55 (3.88) |
|  | Pancreatitis necrotising | 2 | 15.75 (3.93-63.09) | 15.74 (27.55) | 15.71 (4.92) | 3.97 (2.3) |
| Tislelizumab | Immune-Mediated Pancreatitis | 6 | 236.12 (104.92-531.4) | 235.54 (1366.8) | 229.77 (116.55) | 7.84 (6.74) |
|  | Pancreatitis | 2 | 1.39 (0.35-5.57) | 1.39 (0.22) | 1.39 (0.44) | 0.48 (-1.19) |
| Pembrolizumab | Pancreatitis | 141 | 1.5 (1.27-1.77) | 1.5 (23.59) | 1.5 (1.31) | 0.58 (0.34) |
|  | Immune-Mediated Pancreatitis | 84 | 152.86 (117.38-199.07) | 152.77 (8304.95) | 100.52 (80.59) | 6.65 (6.29) |
|  | Pancreatitis Acute | 50 | 1.16 (0.88-1.53) | 1.16 (1.1) | 1.16 (0.92) | 0.21 (-0.19) |
|  | Autoimmune Pancreatitis | 37 | 33.44 (23.8-47) | 33.44 (1044.26) | 30.09 (22.64) | 4.91 (4.42) |
|  | Pancreatitis Chronic | 8 | 1.85 (0.92-3.71) | 1.85 (3.12) | 1.85 (1.03) | 0.88 (-0.08) |
|  | Obstructive Pancreatitis | 8 | 4.92 (2.45-9.9) | 4.92 (24.58) | 4.86 (2.71) | 2.28 (1.31) |
|  | Oedematous Pancreatitis | 2 | 1.8 (0.45-7.24) | 1.8 (0.71) | 1.8 (0.56) | 0.85 (-0.83) |
|  | Pancreatitis Necrotising | 2 | 0.51 (0.13-2.06) | 0.51 (0.91) | 0.52 (0.16) | -0.96 (-2.62) |
|  | Pancreatitis Relapsing | 1 | 0.62 (0.09-4.4) | 0.62 (0.23) | 0.62 (0.12) | -0.69 (-2.73) |
| Nivolumab | Pancreatitis | 244 | 2.09 (1.84-2.37) | 2.09 (136.83) | 2.08 (1.87) | 1.05 (0.87) |
|  | Immune-Mediated Pancreatitis | 62 | 79.26 (59.41-105.74) | 79.23 (3572.32) | 59.35 (46.63) | 5.89 (5.48) |
|  | Pancreatitis Acute | 55 | 1.02 (0.78-1.33) | 1.02 (0.02) | 1.02 (0.82) | 0.03 (-0.36) |
|  | Autoimmune Pancreatitis | 40 | 29.17 (21-40.53) | 29.16 (966.74) | 26.03 (19.77) | 4.7 (4.23) |
|  | Pancreatitis Chronic | 6 | 1.11 (0.5-2.47) | 1.11 (0.06) | 1.11 (0.57) | 0.15 (-0.95) |
|  | Pancreatitis Necrotising | 5 | 1.03 (0.43-2.48) | 1.03 (0) | 1.03 (0.49) | 0.04 (-1.14) |
|  | Obstructive Pancreatitis | 5 | 2.44 (1.01-5.9) | 2.44 (4.22) | 2.43 (1.16) | 1.28 (0.09) |
|  | Pancreatitis Haemorrhagic | 1 | 1.11 (0.16-7.9) | 1.11 (0.01) | 1.11 (0.21) | 0.15 (-1.9) |
| Ipilimumab | Pancreatitis | 95 | 2.87 (2.35-3.51) | 2.86 (115.09) | 2.86 (2.42) | 1.52 (1.22) |
|  | Immune-Mediated Pancreatitis | 21 | 100.1 (63.98-156.59) | 100.05 (1882.02) | 91.52 (62.94) | 6.52 (5.87) |
|  | Autoimmune Pancreatitis | 17 | 47.18 (29.02-76.69) | 47.16 (735.4) | 45.2 (30.1) | 5.5 (4.8) |
|  | Pancreatitis Acute | 12 | 0.82 (0.46-1.44) | 0.82 (0.49) | 0.82 (0.51) | -0.29 (-1.09) |
|  | Pancreatitis Chronic | 1 | 0.66 (0.09-4.7) | 0.66 (0.17) | 0.66 (0.13) | -0.59 (-2.64) |
|  | Oedematous Pancreatitis | 1 | 2.81 (0.39-20.01) | 2.81 (1.16) | 2.81 (0.54) | 1.49 (-0.56) |
