# Supplementary figures and images for: Pancreatitis associated with immune checkpoint inhibitors: a pharmacovigilance analysis based on FDA adverse event reporting system (FAERS) database
Source: Front Pharmacol. 2025 Sep 22;16:1635372. doi: 10.3389/fphar.2025.1635372 (PMC12497980; doi:10.3389/fphar.2025.1635372)

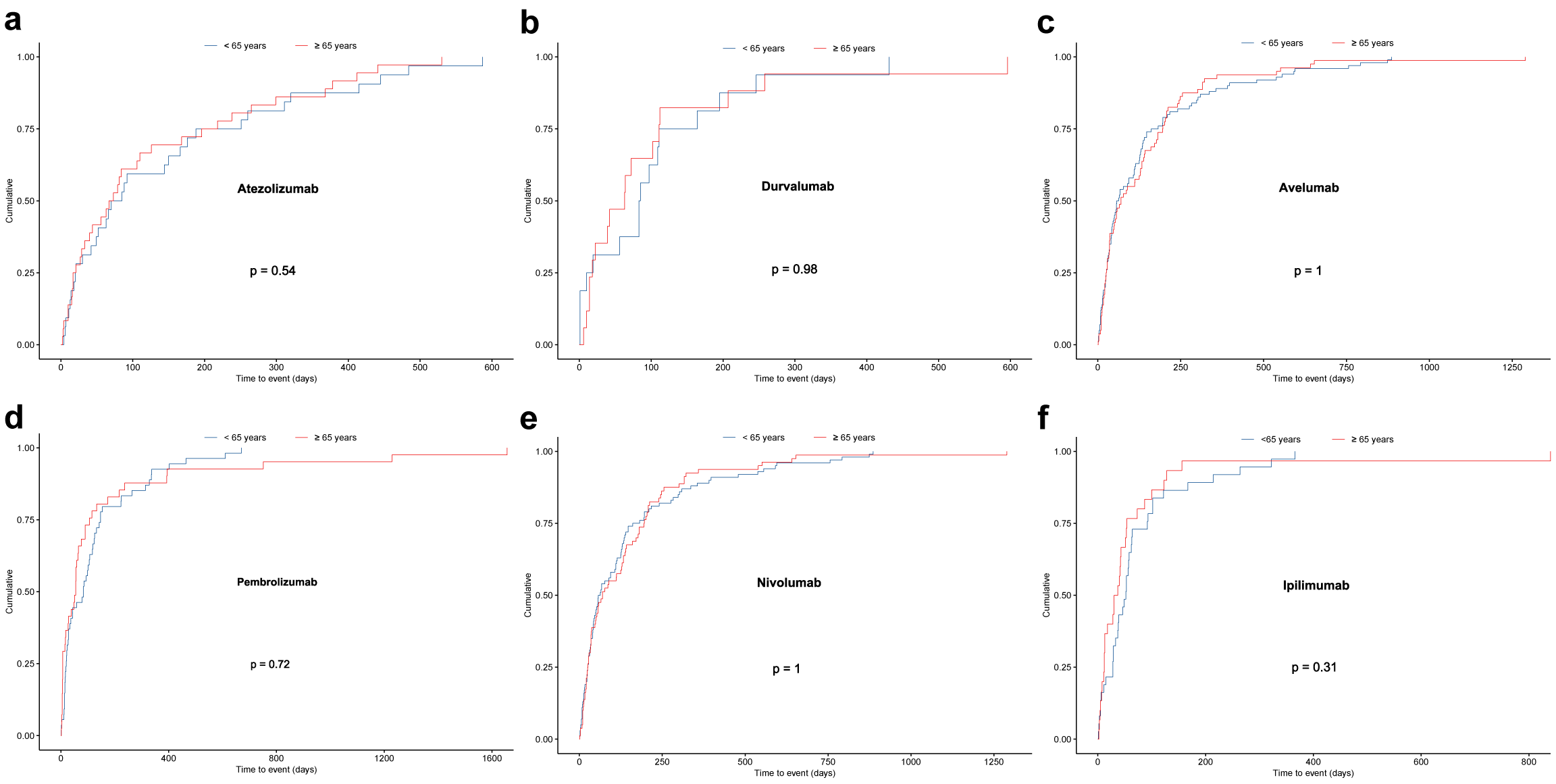

Supplement: Supplementary file 2 [file Image2.tif]

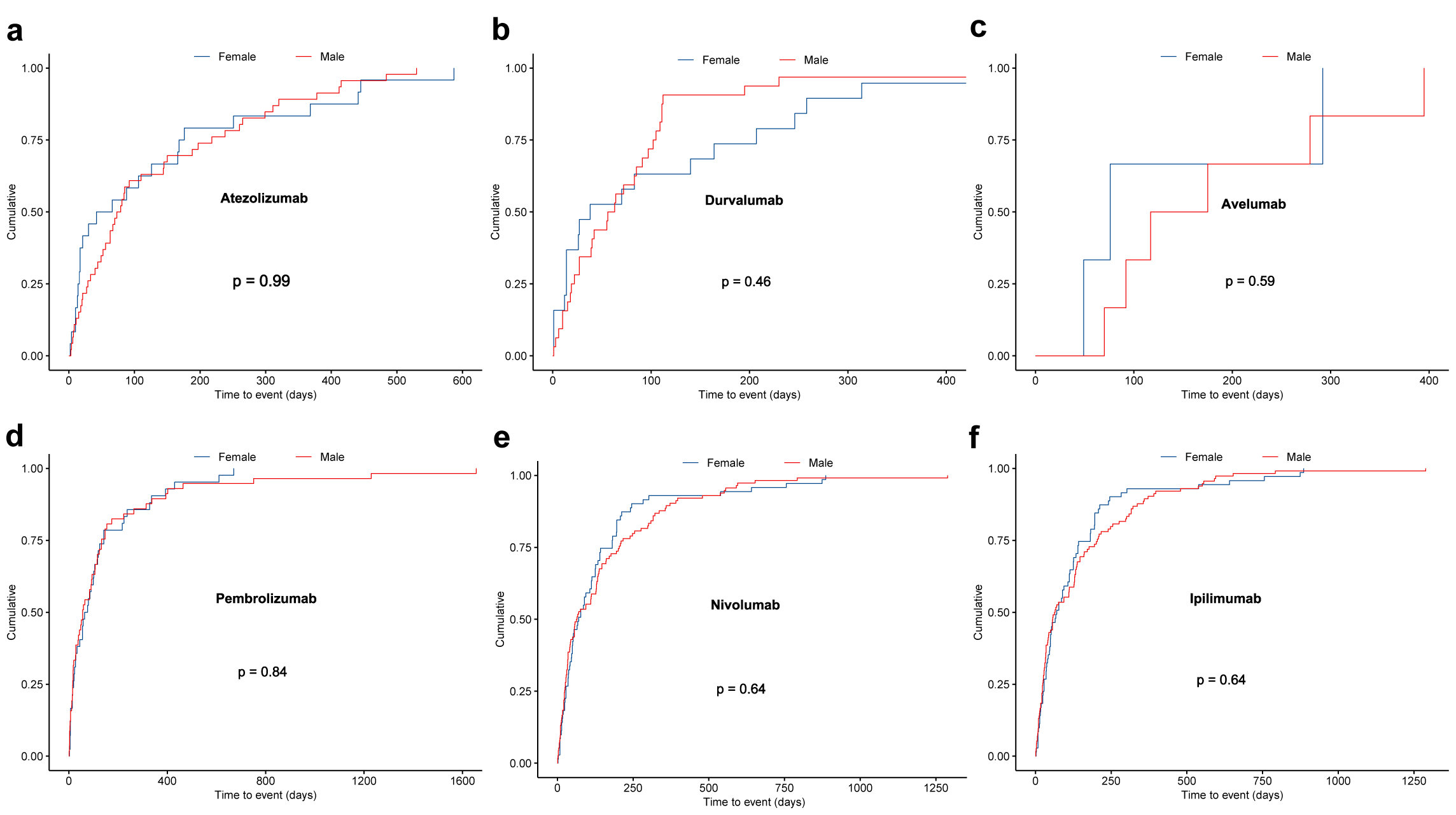

Supplement: Supplementary file 3 [file Image1.tif]
